# The importance of trust-based relations and a holistic approach in advance care planning with people with dementia in primary care: a qualitative study

**DOI:** 10.1186/s12877-018-0872-6

**Published:** 2018-08-16

**Authors:** Bram Tilburgs, Myrra Vernooij-Dassen, Raymond Koopmans, Marije Weidema, Marieke Perry, Yvonne Engels

**Affiliations:** 1 Department of IQ healthcare, Radboudumc, Nijmegen, The Netherlands; 2 Department of Primary and Community Care, Radboudumc, Nijmegen, The Netherlands; 3 Radboudumc Alzheimer Centre, Nijmegen, The Netherlands; 4 Joachim en Anna, Centre for Specialized Geriatric Care, Nijmegen, The Netherlands; 5 Department of Medical Oncology, Radboudumc, Nijmegen, The Netherlands; 6 Department of Geriatric Medicine, Radboudumc, Nijmegen, The Netherlands; 7 Department of Anesthesiology, Pain and Palliative Medicine, Radboudumc, Nijmegen, The Netherlands

**Keywords:** Advance care planning, General practitioner, Dementia, Primary care

## Abstract

**Background:**

ACP enables individuals to define and discuss goals and preferences for future medical treatment and care with family and healthcare providers, and to record these goals and preferences if appropriate. Because general practitioners (GPs) often have long-lasting relationships with people with dementia, GPs seem most suited to initiate ACP. However, ACP with people with dementia in primary care is uncommon. Although several barriers and facilitators to ACP with people with dementia have already been identified in earlier research, evidence gaps still exist. We therefore aimed to further explore barriers and facilitators for ACP with community-dwelling people with dementia.

**Methods:**

A qualitative design, involving all stakeholders in the care for community-dwelling people with dementia, was used. We conducted semi-structured interviews with community dwelling people with dementia and their family caregivers, semi structured interviews by telephone with GPs and a focus group meeting with practice nurses and case managers. Content analysis was used to define codes, categories and themes.

**Results:**

Ten face to face interviews, 10 interviews by telephone and one focus group interview were conducted. From this data, three themes were derived: development of a trust-based relationship, characteristics of an ACP conversation and the primary care setting.

ACP is facilitated by a therapeutic relationship between the person with dementia/family caregiver and the GP built on trust, preferably in the context of home visits. Addressing not only medical but also non-medical issues soon after the dementia diagnosis is given is an important facilitator during conversation. Key barriers were: the wish of some participants to postpone ACP until problems arise, GPs’ time restraints, concerns about the documentation of ACP outcomes and concerns about the availability of these outcomes to other healthcare providers.

**Conclusions:**

ACP is facilitated by an open relationship based on trust between the GP, the person with dementia and his/her family caregiver, in which both medical and non-medical issues are addressed. GPs’ availability and time restraints are barriers to ACP. Transferring ACP tasks to case managers or practice nurses may contribute to overcoming these barriers.

**Electronic supplementary material:**

The online version of this article (10.1186/s12877-018-0872-6) contains supplementary material, which is available to authorized users.

## Background

People with dementia face a progressive decline in functional and mental capacity, with a median survival of 7 to 10 years from the first symptoms of the disease [1–3]. Because of its chronic and life limiting nature and the expected cognitive decline, timely advance care planning (ACP) is advised [4].

A recently published international consensus statement from the European Association of Palliative Care defined ACP as the process which enables people to define goals and preferences for future medical treatment and care, to discuss these goals with family and healthcare providers, and to record and review these preferences if appropriate [5]. Although ACP is recommended by dementia experts, for people with dementia it is uncommon in daily practice and futile medical treatments, avoidable hospitalisations and poor quality of life often occur [2, 4, 6–8].

Research on the effectiveness of ACP for people with dementia is scarce. However, in adult populations, ACP improved the concordance of preferred and delivered care and the communication between patients, their family and healthcare professionals [9, 10]. In frail elderly, ACP reduced anxiety, depression and stress. When ACP was initiated, frail elderly also received less aggressive treatments, were less often admitted to the hospital and more often died in their trusted environment [11]. Dementia-specific research in long-term care settings showed that ACP reduced healthcare costs and hospital admissions [12].

Compared to people with dementia who are institutionalized, community-dwelling people with dementia more often have the mental capacity to express their preferences for future care and to actively participate in ACP. In the Netherlands, over two third of the people with dementia live in the community with general practitioners (GP), often assisted by a practice nurse, as primary healthcare providers [13]. In many cases, case managers are also involved to coordinate different aspects of care and provide emotional support [14]. Because most people with dementia and their family caregivers have long-lasting relationships with their GPs, GPs seem suited and willing to initiate ACP [15, 16]. In primary care however, ACP with people with dementia hardly takes place or takes place very late [12, 17, 18].

Previous research identified uncertainties about the timing, future, evaluation and decisional capacities of people with dementia to contribute to the limited initiation of ACP [19]. A timely start, facilitates ACP, because in the beginning of the disease process when cognitive decline is still mild, participating in decision making is still possible. Involving people with dementia and family caregivers and regularly reviewing and documenting ACP outcomes facilitates ACP as well [19].

Because of the difficult subjects being discussed, it can be assumed that the communication and relationship with the GP are also important facilitators for ACP [20]. Dementia-specific knowledge about this topic is however limited [21, 22]. Previous research also showed that people with dementia favour discussing non-medical issues within ACP [23]. This holistic approach were the psychological, social and spiritual domains, next to the physical domain are included, fits the definition palliative care and the broad definition of ACP as proposed by Rietjens et al. [5, 24]. Evidence on this potential facilitator is however also limited.

As ACP with people with dementia by GPs is still rarely practiced, we aimed to further explore barriers and facilitators concerning this subject. We thereby especially focused on the evidence gaps concerning the communication between the GP and people with dementia, the relationship of GPs with people with dementia and the inclusion of non-medical preferences within ACP.

## Methods

### Research design

A qualitative design was used in order to reach our research aim [25]. We included people with dementia, living independently in the community or a in a residential home and receiving care from a GP, together with their family caregivers. GPs, practice nurses and case managers were included because they are important stakeholders in the care for people with dementia [26].

Case managers and practice nurses were interviewed during a focus group, which method is particularly useful to explore the participants’ knowledge and experiences [27]. Because of their busy time schedule, GPs were interviewed by telephone, as this facilitated flexibility in scheduling the interview. People with dementia, together with their family caregivers, were interviewed face to face in their own homes, as their cognitive decline might impede group discussions.

### Recruitment of participants

We recruited GPs by contacting the GP peer review group of the department of primary and community care of the Radboudumc and through the professional contacts of the researchers involved in our study (MP, BT and YE). We strived for a sample of GPs which contained males and females and a variety of experience with dementia care, as both characteristics influence general practitioners’ attitudes towards dementia [28].

People with dementia, their family caregivers, case managers and practice nurses were recruited during several community meetings for people with dementia and family caregivers (Alzheimer café’s) in the region of Nijmegen and through the professional contacts of one of the researchers (MP). We decided to interview people with dementia accompanied by their family caregivers because earlier research showed that they prefer making decisions about future care together [23, 29]. Furthermore, we considered it very important for participating people with dementia to feel safe discussing such a delicate topic. GPs and case managers and practice nurses could participate if they were involved in the care for people with dementia. Potential participants were informed by letter about the study and were requested to sign an informed consent before the interview.

### Data collection

The main researcher (BT), a male PhD candidate, psychologist and nurse, trained in conducting and analysing qualitative interviews, was present during all interviews. Two additional interviewers were: a female researcher in palliative care, trained in conducting and analysing qualitative interviews (YE) and a female medical student, with no prior experience in qualitative research (MW). No relationship existed with the respondents prior to the interviews.

The interviews with the GPs were conducted by one researcher (BT). The face to face interviews with people with dementia and family caregivers were conducted by two researchers (BT, MW) as was the focus group (BT, YE). Field notes were made during each interview and participants gave their consent to audio-tape the interviews.

A similar topic guide was used for all three forms of interviewing. This guide was developed during several sessions with the members of the research team (BT, MP, YE, MVD, RK) and pilot tested with a family caregiver and a person with dementia (Additional file 1). All people with dementia and family caregivers received a written summary of their interview and were invited to give comments.

### Data analysis

All interviews were transcribed verbatim. Data analysis started directly after the first interview using content analysis [30]. After each interview had been coded, the topic list was adapted where required. Researchers independently open coded all interviews within each group of stakeholders (people with dementia/family care givers: BT and MW; GPs/case managers and practice nurses: BT and EB or RT or PL). Results were compared until consensus was reached. In case of disagreement, this was discussed with a senior researcher (YE, MP). After the last interview within each group of stakeholders, the researchers made an affinity diagram to cluster codes and define categories and themes [30, 31]. All data were then combined to create definitive categories and themes. Because we wanted to focus on new findings, codes already thoroughly described in earlier research were marked. The codes concerning new findings will be described in the results section. The codes already known from earlier research will only be presented in a table.

### Ethical consent

The study was approved by the research ethics committee (CMO) of the region Arnhem-Nijmegen in accordance with the Medical Research Involving Human Subjects Acts and the declaration of Helsinki (NL52613.091.15). Anonymity was assured by removing all participant information that could lead to identification from the transcripts.

## Results

GPs aged 31 to 64 years and their work experience varied between one to 33 years. Sixty percent of the GPs was female. The number of patients within each practice ranged between 1700 and 7370 and the percentage of persons with dementia in their practice varied between 0.1 and 10%. One GP was trained as an expert GP elderly care. Case managers/practice nurses aged 46 to 63 years. Their work experience ranged between 5 and 25 years with a case load between 55 and 75 people with dementia. All were female and trained in dementia care. People with dementia aged 79 to 90 years and 70% was male. The time since diagnosis ranged from 6 months to 6 years. Family caregivers aged 24 to 85 years and 77% was female. Nine people with dementia lived in their own home with their family caregiver. One lived in a residential home, separately from her family caregiver.

One focus group of 90 min with case mangers and practice nurses was conducted. The interviews by telephone with GPs lasted 30 min. The interviews with people with dementia and family caregivers lasted 90 min. With the GPs, people with dementia and family caregivers, no new codes emerged after eight interviews. To confirm saturation two additional interviews were conducted. In three interviews with people with dementia and their family caregivers, an extra family caregiver was present. One person with dementia passed away after we already made the appointment for the interview. Because the widow of this person explicitly asked to participate and seemed capable to express her husband’s view as well as her own, we decided to keep her included in this study. Field notes were made during each interview and participants gave their consent to audio-tape the interviews.

Content analysis revealed barriers and facilitators for initiating ACP by GPs with people with dementia in three themes: development of a trust based therapeutic relationship, characteristics of an ACP conversation, the primary care setting and eight categories: the relationship with the general practitioner, home visits, starting ACP, stakeholder involvement, discussing goals, evaluation and documentation, time availability, organisation of the general practice. These themes, categories and codes are displayed in Table 1.

**Table 1:** Themes, categories and codes

Themes	Categories	Codes	
		Facilitators	Barriers
Development of a trust based therapeutic relationship	The relationship with the GP	The GP knows what PWD find important in life (PWD)	The GP is to distant (FC,PWD)
		The GP is easy to talk to (PWD)	The GP does not listen to PWD (FC)
		An open relationship with the GP is important (PWD)	The GP has little contact with PWD (PWD, FC,GP, CM)
		A trusting relationship with the GP is important (CM, FC, PWD, GP)	The GP trivialises the situation (PWD, FC)
		The GP listens to the PWD (PWD, FC)	
		The GP knows the PWD/FC personally (PWD, FC, GP)	
		The GP provides empathic support (FC, PWD, GP)	
		The GP understands the PWD (PWD)	
		Providing information respectful is important (PWD, GP)	
		*The GP provides the right information (PWD)*^a^ [52]	
		*Good communication makes ACP easier (GP)*^a^ [21, 22]	
		*A good relationship with the GP is important (PWD, FC)*^a^ [21]	
	Home visits	ACP should take place at home (CM, FC, PWD)	The GP does not conduct home visits(FC, PWD)
		ACP should take place at a quiet moment (FC, PWD)	The GP does not know the living situation (CM, FC)
		More time available during home visits (FC)	
		By conducting home visits, the GP knows the living situation (CM, FC)	
		ACP should be held at the PWD’s preferred location (GP)	
Characteristics of an ACP conversation	Starting ACP	ACP starts after providing the diagnosis (GP)	Not all PWD/FC want ACP (PWD, GP)
		ACP should not start under stress (CM, GP)	GP’s lack knowledge/experience of ACP (GP)
		PWD/FC should first cope with the diagnosis before the start of ACP (GP)	The diagnosis is not always clear (GP)
		ACP should start when the PWD/FC states the need to do so (GP)	GP doesn’t take the initiative to start ACP (CM, FC, PWD)
		FC takes the initiative to start ACP (FC)	Healthcare professionals find discussing end of life issues difficult (CM)
		Because of a wish for euthanasia, ACP is started (PWD)	Dementia does not give complaints (PWD)
		PWD must be followed from diagnosis on (GP)	Start ACP when problems arise (CM, GP, PWD, FC)
		Information from family and healthcare providers stimulates the start of ACP (GP)	*The assessment of decisional competency is difficult*^a^ [46]
		Surprise Question helps to start ACP (GP)	
		*ACP should start early because of the cognitive decline (GP, FC, PWD, CM)*^a^ [21, 22, 45–47]	
		*GPs should take the initiative for ACP (GP, CM, PWD, FC)*^a^ [16, 21, 22]	
		*The GP’s positive attitude stimulates the start of ACP (GP)*^a^ [22]	
	Stakeholder involvement	Provide choices instead of open questions (GP)	ACP is confronting for PWD (GP)
		ACP should not be confronting (GP)	Religion limits discussions about future care (GP)
		ACP content must be adjusted to PWD level of understanding (FC, GP)	Social status influences ACP (GP)
		All healthcare providers should be present during ACP (GP)	PWD’s/FC’s IQ and self-knowledge influences ACP (GP)
		ACP with the FC and GP without PWD sometimes takes place (FC)	Multiple healthcare providers present during ACP limits ACP (GP)
		*End of life decisions are made together (FC, PWD)*^a^ [45, 53, 54]	Preferences of FC and PWD can differ (CM, GP)
		*FC must present within ACP (CM, FC, PWD, GP)*^a^ [45, 53, 54]	ACP is difficult to explain (GP)
		*FC makes ACP decisions (PWD, FC)*^a^ [45, 53]	*The assessment of decisional competency is difficult (GP)*^a^ [46]
		*PWD must be present when ACP is discussed (GP, FC, CM, PWD)*^a^ [45, 53–55]	
Characteristics of an ACP conversation	Discussing goals	PWD’s preferences are the starting point of ACP (GP CM)	Not all problems can be discussed upfront (GP)
		FC respects PWD choices (FC)	
		PWD/FC want to be able to prepare ACP (CM, PWD, FC)	
		ACP decisions provide clarity and peace (FC, PWD, GP)	
		The GP sometimes must be authoritarian (GP)	
		ACP should deal with current issues (GP)	
		Supporting FCs should be discussed during ACP (FC)	
		Medical subjects should be discussed during ACP (CM, PWD,FC)	
		social subjects should be discussed during ACP (PWD,FC)	
		PWD know what they want for their future (FC, PWD)	
		ACP prevents moments of crisis and over treatment (GP)	
		ACP stimulates autonomy (GP)	
		Through ACP the GP can explain care possibilities (GP)	
	Evaluation and documentation	ACP should not be evaluated to often (CM)	ACP documentation not always available for all stakeholders (GP, FC, PWD, CM)
		*ACP must be evaluated regularly (GP)*^a^ [45, 54]	ACP decisions are considered final (FC)
		*ACP outcomes must be documented and available for all stakeholders (GP, CM)*^a^ [21, 45–47]	The PWD’s current will counts (CM, GP, FC)
		*ACP must be a cyclical process (PWD,FC,CM, GP)*^a^ [45, 54]	*When to evaluate ACP is unclear (GP)*^a^ [54]
The primary care setting	Time availability	The GP should take enough time for ACP (FC)	ACP consultations are often to short (GP, MC, PWD, FC)
		The GP is easily available (FC)	GP has limited time for ACP (FC)
		ACP saves time in the long term (GP)	Because of limited time only medical subjects are discussed (PWD, FC, CM)
			The GP is rushed during ACP (FC)
			ACP doesn’t save time in the long term (GP)
			ACP takes time in the short term (GP)
			Planning an ACP conversation is sometimes difficult (GP)
	Organisation of the general practice	regular appointments with GP/CM/PN facilitates ACP (FC, PWD, GP)	Casemanager is often involved to late (GP, CM, PWD, FC)
		CM/PN discusses medical and social subjects (FC)	PWD have limited contact with their CM/PN (FC)
		CM/PN has more knowledge of living situation compared to GP (FC,GP, PWD)	PN/CM cannot discuss medical issues (GP)
		CM/PN has more knowledge of dementia compared to GP (CM, PWD)	Inadequate reimbursement limits ACP (GP)
		The therapeutic relationship with the CM/PN facilitates ACP (PWD, FC)	
		ACP can also be provided by a CM/PN (FC)	
		GPs and CMs/PNs should have regular contact (FC, GP)	
		Specialized training in dementia/elderly care stimulates ACP (GP)	
		PN/CM can support GP in ACP process (GP)	
		GP should coordinate ACP (GP)	
		Special care programs for dementia facilitate ACP (GP)	
		ACP should be structurally implemented (GP)	

*GP* stated by general practitioner, *CM* stated by casemanager/practice nurse, *PWD* stated by person with dementia, *FC* stated by family caregiver

^a^codes which already have been described in earlier research

Several codes within the categories of the relationship with the GP, starting ACP, stakeholder involvement and evaluation and documentation were identical to barriers and facilitators described in earlier research (Table 1) and were therefore not described in the result section.

### Theme 1: Development of a trust based therapeutic relationship

#### The relationship with the general practitioner

##### Facilitators

GPs, people with dementia and family caregivers stated that it is important that the GP knows the person with dementia personally, is empathic, supportive and provides information respectfully. People with dementia and their family caregivers added that, when discussing preferences for the future, they want their GP to listen to them, is easy to talk to and knows what they find important in life.

*“A connection, an invisible connection, but that is a feeling, a feeling you have that you are at ease because she (GP) is there. He (person with dementia) did not have to be afraid anymore. He did not have to worry. He did not have to be nervous If he couldn’t remember something, well... he could get his thoughts of his mind so to speak..... There was a trusting relationship which was beautiful to see” (family caregiver, interview 2).*

##### Barriers

Several family caregivers and people with dementia stated that their GP trivialized their situation, was too distant and did not listen to them. This made them hesitant to discuss sensitive topics. GPs, people with dementia, family caregivers and case managers and practice nurses also found that GPs had too little contact with people with dementia and their family caregivers. According to some GPs, having infrequent contacts was due to either a capable family caregiver or the person with dementia living in a residential home.

#### Home visits

##### Facilitators

People with dementia, family caregivers, case managers and practice nurses preferred to have ACP at the home of the person with dementia. In this trusted environment, people with dementia are more at ease to talk about sensitive topics and feel less hurried by the GP’s time schedule. People with dementia, family caregivers, case managers and practice nurses found that during such home visits, GPs get more insight in the person with dementia’s living situation.

*“I would prefer to have the conversation here (at home) and not at an impersonal office. The home environment is different; than you can sit in your own chair and communicate about personal topics” (family caregivers, interview 9).*

According to the GPs, ACP conversations ideally should take place at the location preferred by the person with dementia and family caregiver.

##### Barriers

Case managers and practice nurses and GPs doubted if home visits for ACP would be feasible because of the GP’s busy schedule. This corresponded with the fact that, according to most people with dementia and family caregivers their GPs rarely conduct home visits.

### Theme 2: Characteristics of an ACP conversation

#### Starting ACP

##### Facilitators

Some GPs, people with dementia, family caregivers and all case managers and practice nurses wanted to start ACP immediately after the diagnosis was given. Starting early has the advantage of being able to choose the moment of initiation and ACP under stressful circumstances can be avoided.

*“Yes, uh.... it also depends on the co-morbidities but for me pretty soon... That is difficult because what is pretty soon... But I would say that from diagnosis, you want to start to discuss what peoples wishes are.....” (general practitioner, interview 2).*

Other GPs also stated that dyads should first be given time after the disclosure of the diagnosis, because this is often a difficult experience. Case managers and practice nurses, people with dementia and family caregivers added that people with dementia and family caregivers should be given time before an ACP conversation to think about what they want to discuss.

According to several GPs, some types of information received from other healthcare professionals or family caregivers could trigger them to start ACP. According to some people with dementia, ACP is initiated sooner when a wish for euthanasia was expressed. One GP added that using the Surprise Question (a question the general practitioner asks himself in silence to identify those patients with an increased chance to die or deteriorate within a year) stimulated his proactive behaviour.

##### Barriers

Part of the GPs, people with dementia and family carers only wanted to discuss preferences for the future when problems actually arise. Some GPs said to postpone ACP until the cognitive deterioration becomes problematic, and for that reason monitored the person with dementia after the diagnosis had been provided. A lack of knowledge and experience with ACP, an unclear diagnosis and the fact that ACP is a difficult concept to explain were other reasons to postpone ACP, as mentioned by GPs. Finally, according to case managers and practice nurses, GPs do not initiate ACP as they fear talking about difficult subjects.

#### Stakeholder involvement

##### Facilitators

Some GPs wanted all healthcare professionals involved in the care for a person with dementia to participate in ACP so that all knew what had been discussed and decided. Some GPs also stated that, if the person with dementia approved, ACP consultations sometimes took place without the person with dementia. For example, when the person with dementia denied or did not accept the dementia diagnosis. Family caregivers and GPs found that, in order to stimulate involvement of people with dementia, the GP should tailor ACP to the cognitive level of the person with dementia and make sure that the conversation is not confronting. When GPs asked closed instead of open questions, participation of people with dementia within ACP also becomes easier.

##### Barriers

Some GPs mentioned that people with dementia’s and family caregivers’ low social status, low IQ, limited self-knowledge or strong religious beliefs sometimes made involving dyads in ACP difficult. According to some GPs, the presence of multiple family caregivers during ACP was a disturbance and therefore only wanted ACP with the person with dementia and their family caregiver.

#### Discussing goals

##### Facilitators

 According to GPs, case managers and practice nurses, people with dementia’s life values, wishes and goals must be the starting point of ACP and such a conversation should therefore begin with what they find important in life. People with dementia, family caregivers, case managers and practice nurses explicitly mentioned that during ACP not only medical (e.g. do not resuscitate statements, hospital admissions) but also non-medical subjects (e.g. daytime activities, social contacts, what bothers him or her at this moment) should be discussed. Case managers and practice nurses, people with dementia and family caregivers agreed that if people with dementia express a wish for euthanasia, this topic should be addressed as well.

*“We discussed the human aspect..........but also if we can still keep on living in this house and if more care has to be provided. He (person with dementia) doesn’t want to move........ He himself is the driving force behind this (ACP). He wants to anticipate...” (family caregiver, interview 8).*

The discussion of goals had additional advantages. According to GPs, it gave them the opportunity to explain possible care options and in addition provide clarity, peace, stimulate mourning and prevent overtreatment, which often happened when decisions had to be made all of a sudden in moments of crisis. Some GPs added that discussing goals fostered autonomy. However, sometimes a paternalistic approach was found necessary.

##### Barriers

If the preferences of people with dementia and family carers differed, GPs, case managers and practice nurses found the discussion of goals more difficult. GPs also expressed that it is not always possible to openly discuss all potential future problems.

#### Evaluation and documentation

##### Facilitators

In the opinion of case managers and practice nurses, reviewing ACP too often made it seem artificial. In their opinion, reviewing ACP every 6 to 12 months was sufficient.

##### Barriers

Most family caregivers and GPs agreed that the current wishes of people with dementia should be leading, even if this would contradict earlier decisions. One family caregiver considered ACP decisions to be binding and therefore found ACP evaluation unnecessary.

People with dementia, family caregivers, GPs and case managers and practice nurses doubted if important ACP outcomes are documented in a structured way by GPs and raised concerns about the availability of such outcomes for other healthcare professionals.

### Theme 3: The primary care setting

#### Time availability

##### Facilitators

According to family caregivers, the GP should be easily available and should take enough time for ACP consultations. According to part of the GPs, although ACP requires short term time investments, it saves time in the long run.

##### Barriers

All interviewees found the usual duration of a consultation too short for ACP. Consequently, according to people with dementia, family caregivers and case managers and practice nurses, GPs mainly address medical subjects and are rushed during ACP.

*“When we came in, the first thing she (the GP) said was; I don’t have much time and then she said: good afternoon. She sat down and started to fire all sorts of questions at my mother. My mother didn’t know what was going on... At a certain moment I said; stop!.... This doesn’t make her happy at all.” (family caregiver, interview 9).*

According to part of the GPs, ACP demands short term time investments while time is scarce and they were not convinced that ACP would save time in the long term. Moreover, some GPs stated that they do not have time to plan and regularly review ACP decisions.

#### Organisation of the general practice

##### Facilitators

GPs found guidelines and the use of specialised care programs for dementia useful. These helped them to initiate ACP in a structured manner. The involvement of a case manager and practice nurse could further accommodate ACP. According to case managers and practice nurses and family caregivers, case managers and practice nurses have more knowledge of dementia. They also have more time to plan, prepare and carry out ACP than GPs do. Case managers and practice nurses, compared to GPs, also have more opportunities to monitor people with dementia and family caregivers and thereby identify problems early.

*Quote: “We use a certain score and when somebody is frail,......our practice nurse visits them at home.... more in a general sense.... to see...... how are you doing? What are the problems now, but also what do you expect in the future?” (general practitioner, interview 6).*

People with dementia and family caregivers confirmed this and added that regular contact and the therapeutic relationship they develop with a case manager and practice nurse helped them to discuss preferences for future care.

##### Barriers

Some GPs stated that case managers and practice nurses cannot make medical decisions and therefore can only partly conduct ACP. Some GPs stated that they occasionally forgot to make use of a case manager and practice nurse. This was confirmed by case managers and practice nurses who as a result were often involved late.

Difficulties with reimbursing ACP within the Dutch healthcare system were also mentioned as a problem by GPs. Solving this would stimulate them to initiate ACP, also because some believe that ACP will reduce healthcare costs.

## Discussion

In this study we aimed to further explore barriers and facilitators for ACP with people with dementia by GPs, with a focus on the evidence gaps concerning the communication between the GP and people with dementia, the relationship of GPs with people with dementia and the inclusion of non-medical preferences within ACP. Newly found facilitators are: having a relationship with the GP that is built on trust and mutual understanding, and the discussion of ACP in the comfort of people with dementia’s homes. Explicitly addressing non-medical issues in ACP discussions, with a focus on discussing people with dementia’s current and short-term goals was considered a facilitator by all stakeholders. The involvement of a case managers and practice nurse also facilitates ACP. GPs’ lack of time is an important barrier for ACP. To two other barriers known from earlier research nuances could be added: Some participants wanted ACP to start early, while others wanted to wait until problems actuality arise. Stakeholders raised concerns about the availability of ACP documentation to all professionals involved. They were willing to review ACP but not often.

The importance of the relationship with the GP, as stressed by the participants in this study, is in line with earlier research in primary care [32]. Patients who suffer from more severe diseases or who have problems with psychosocial or existential impact, such as people with dementia, appraise their relationship with the GP as more important [33]. Unfortunately, the focus during consultations still seems to be on treatment compliance with little attention to social, psychological or spiritual issues, even when advance directives are discussed [34, 35]. Particularly in dementia, with its high psychosocial and existential burden and specific relational and communicational needs, this can be considered an omission [3, 36, 37].

As shown in our results and in a systematic review on GP communication, home visits and taking time are important when difficult subjects are discussed [38]. When GPs take more time, more psychosocial problems are attended and patient satisfaction rises [39]. However, it is also known that GPs are busy, and time per consultation is limited. This time restraints seems an important reason for the limited number of home visits, for the GPs’ main focus on medical problems and for the inadequate assessment of care needs [40–42]. The lack of ACP by GPs therefore seems, at least partly, caused by how GPs are organized.

### Recommendations for future practice

Participants in our study mentioned the use of case managers or practice nurses as a possible solution for overcoming problems concerning the development of a therapeutic relationship and the available time. Case managers and practice nurses have more opportunities to visit people with dementia and thereby develop a therapeutic relation which seems so important [43]. When using collaborative care models, in which case managers or practice nurses take on certain tasks of the GP, regular consultations between GPs and case managers and practice nurses are advised and division of tasks regarding ACP should be explicitly addressed [44]. This facilitates a combined medical, psychosocial and spiritual and thus holistic approach. Also, when multiple disciplines are involved, it is essential that preferences for future care are clearly documented and made available to all [21, 45–47]. Because case managers and practice nurses also have time constraints their caseload must be monitored [43].

In recent years ACP has shifted from a document driven conversation where mainly options for medical treatments and end-of-life preferences were discussed, to a broader scope where the physical, psychosocial and spiritual domains are all included [5, 48]. Previous research and this paper showed that a broad approach to ACP including non-medical issues is a facilitator for people with dementia. When their valued abilities or activities are used to justify their choices, their participation in ACP can be established despite of their cognitive decline [29]. This holistic approach which starts with the person’s with dementia current wishes and concerns, therefore contributes to their autonomy and can also be used to guide further decision making about future care [49, 50]. ACP then is extended to something more than just a ‘checkbox’ for medical decisions. It becomes an open encounter between people with dementia, family caregivers and healthcare professionals during which a wide array of preferences concerning future care can be discussed that may contribute to living well with dementia [51].

### Strengths and limitations

The inclusion of all important stakeholders involved in ACP with people with dementia in primary care is the main strength of this study. By integrating the findings from different perspectives, robust and all-embracing insights were built [25]. As we chose to interview people with dementia and family caregiver dyads in their own homes, we were able to discuss delicate topics in their trusted environment without any rush or disturbance. This gave the participants the opportunity to provide in depth information which enriched our data and conclusions.

The study also has some limitations. Because of recruitment difficulties, the number of case managers and practice nurses participating in this is study was limited and we were not able to conduct ideal purposive sampling. As a result, some beliefs or experiences may not be represented in our data [25]. However, as we reached saturation in the interviews with people with dementia, family caregiver dyads and GPs, and no new themes emerged within the focus group with case managers and practice nurses, we assume that all themes concerning our research aim were exposed.

The fact that we choose to conduct the interviews with people with dementia accompanied by their family caregiver may have influenced our study outcomes. As our results show, their preferences for future care sometimes differ. For that reason both parties may possibly have expressed some different views when interviewed alone. This therefore might be addressed during future research. However, our interview strategy is similar to the situation in daily practice, in which GPs usually discuss future care with patients and informal caregivers together.

Our study outcomes may also be influenced by the specific region in which we conducted our research. This may therefore also be addressed during future research.

## Conclusion

When people with dementia and family caregivers discuss preferences for future care with their GP, home visits, an open relation built on trust and addressing non-medical issues, particularly those in the near future, are key facilitators to ACP.

GPs’ busy time schedule is an important barrier. Case managers and practice nurses have more opportunities to regularly conduct home visits, gain insight in the living situation and to start an open trust-build relationship with people with dementia and their family caregivers. This provides them with the opportunity to use a goal-oriented approach and discuss a broad range of topics. Collaborative care models might therefore help to overcome the time barrier and contribute to exploiting the newly found facilitators to ACP and contribute to living well with dementia.

## Additional file

Additional file 1: Topic guide: barriers and facilitators for GPs when discussing ACP with people with dementia. The topic guide used when interviewing GPs, case managers, practice nurses, people with dementia and family caregivers on barriers and facilitators of ACP with people with dementia by GPs. (DOCX 12 kb)

## Abbreviations

ACP: Advance care planning
GP: General practitioner
