# Supplementary material for: The importance of trust-based relations and a holistic approach in advance care planning with people with dementia in primary care: a qualitative study
Source: BMC Geriatr. 2018 Aug 16;18:184. doi: 10.1186/s12877-018-0872-6 (PMC6097199; doi:10.1186/s12877-018-0872-6)
Supplement: Supplementary file 1 — Topic guide: barriers and facilitators for GPs when discussing ACP with people with dementia. The topic guide used when interviewing GPs, case managers, practice nurses, people with dementia and family caregivers on barriers and facilitators of ACP with people with dementia by GPs. (DOCX 12 kb) [file 12877_2018_872_MOESM1_ESM.docx]

**Topic guide: barriers and facilitators for GPs when discussing ACP with people with dementia.**

Can you tell something about an advance care planning conversation you had?

- What went well, what could be improved?

What, in your opinion, should the role of the GPs be when preferences for future care are discussed?

- Who should take the initiative ?
- Who should be present during ACP ?
- When should such an conversation take place ?
- Do other disciplines, besides the GP, have a role in ACP as well ?
- Are there important (relational)-aspects when discussing ACP ?

Which subjects should be discussed during advance care planning ?

- Are there specific subjects you do or do not want to discuss ?
- How do you think ACP conversations should be documented ?
- When and how often, do you think ACP should be reviewed ?
